# Interplay of physical activity and sleep in modulating heart rate variability: insights from adults with major depressive disorder

**DOI:** 10.3389/fphys.2026.1886722

**Published:** 2026-07-01

**Authors:** Arijita Banerjee, Sumit Kumar

**Affiliations:** 1 Department of Physiology, Indian Institute of Technology Kharagpur, Kharagpur, India; 2 Department of Psychiatry, DRIEMS Medical College, Cuttack, India

**Keywords:** autonomic nervous system, cardiovascular risk, DSM-5-TR, heart rate variability, major depressive disorder, PHQ-9, physical activity, Pittsburgh sleep quality index

## Abstract

**Background:**

Major depressive disorder (MDD) is a leading contributor to global disability and is increasingly recognised as a significant cardiovascular risk factor mediated through autonomic nervous system (ANS) dysregulation.

**Methods:**

This cross-sectional comparative study investigated associations between sleep quality, physical activity, and heart rate variability (HRV) including Poincare plot indices (SD1, SD2, SD1/SD2 ratio) in 46 adults with Diagnostic and Statistical Manual of Mental Disorders (DSM-5-TR) confirmed MDD versus 46 non-depressed controls (aged 18–45 years) recruited at the Dr BC Roy Multi-Speciality Medical Research Centre, IIT Kharagpur, India.

**Results:**

MDD participants demonstrated markedly reduced HRV across all domains: SDNN (39.2 ± 14.8 vs 58.0 ± 18.6 ms), rMSSD (27.4 ± 12.6 vs 46.2 ± 15.4 ms), SD1 (19.4 ± 8.8 vs 32.6 ± 11.2 ms), SD2 (52.2 ± 18.6 vs 77.4 ± 21.8 ms), and high-frequency power (5.36 ± 0.88 vs 6.56 ± 0.80 ln ms²; all p < 0.001). Poor sleep quality (PSQI ≥ 7) was significantly more prevalent in MDD (82.6% vs 47.8%). PSQI scores were independently associated with reduced HRV in multivariable regression, with associations substantially stronger in MDD participants and particularly in females with MDD. Physical inactivity amplified sleep-related HRV decrements.

**Conclusion:**

These cross-sectional findings are consistent with the hypothesis that comprehensive HRV assessment, systematic sleep quality screening, and structured physical activity promotion may be beneficial components of MDD management, pending confirmation from longitudinal interventional studies.

## Introduction

Major depressive disorder (MDD) affects approximately 280 million people worldwide and ranks among the foremost contributors to global disability (Kumar and Banerjee, 2026). Beyond its neuropsychiatric burden, MDD is increasingly recognised as an independent cardiovascular risk factor: individuals with depression demonstrate a two- to fourfold elevated risk of cardiac mortality relative to non-depressed populations (Kumar and Banerjee, 2023). A central mechanistic pathway linking MDD to cardiovascular disease involves dysregulation of the autonomic nervous system (ANS), specifically disrupted sympatho-vagal balance reflected in attenuated heart rate variability (HRV) (Vogelzangs et al., 2010).

HRV, the temporal variation between successive R-R intervals on the electrocardiogram, provides a validated, non-invasive index of cardiac autonomic regulation. Reduced HRV indicates heightened sympathetic dominance, impaired parasympathetic modulation, and elevated risk of arrhythmia, sudden cardiac death, and all-cause mortality (Pavlov and Tracey, 2012). In MDD, chronic autonomic imbalance suppresses parasympathetic outflow and amplifies sympathetic drive, resulting in reduced HRV; proposed mediating pathways include hypercortisolemia and systemic neuroinflammation, each of which may impair vagal activity (Hartmann et al., 2019).

Complementary to conventional time-domain and frequency-domain HRV metrics, Poincare plot analysis, a nonlinear geometric approach, confers additional mechanistic insight. The SD1 index reflects instantaneous beat-to-beat HRV and predominantly indexes parasympathetic activity, while SD2 captures longer-term HRV encompassing both branches of the ANS. The SD1/SD2 ratio characterises the relative balance of autonomic contribution (Banerjee, 2026).

Sleep disturbances are near-universal in MDD, occurring in 70–90% of individuals during active depressive episodes (Liu et al., 2023). MDD is characterised not only by disrupted sleep architecture but by broader dysregulation of biological rhythms, including blunted circadian amplitude and delayed phase, which independently contribute to autonomic instability (Guerrera et al., 2025). Poor sleep quality has been associated with sympatho-vagal imbalance, elevated cortisol secretion, and pro-inflammatory cytokines that may further impair cardiac autonomic regulation. Conversely, adequate physical activity has been associated with improved parasympathetic tone through baroreflex sensitisation and circadian stabilisation, modulating both sleep quality and autonomic function (Stapelberg et al., 2012).

Despite growing recognition of the sleep–autonomic–mood triad, very few studies have simultaneously examined sleep quality, physical activity, and a comprehensive HRV panel incorporating Poincare analysis in MDD populations, particularly in South Asian contexts, where several risk profiles diverge substantially from Western cohorts: population-level physical inactivity rates are substantially higher, dietary patterns and obesity phenotypes differ, sociocultural stigma around mental health limits help-seeking and may prolong untreated depressive episodes, and epidemiological data indicate that South Asians carry disproportionate cardiovascular risk at lower BMI thresholds, all factors that may amplify the sleep–autonomic–mood triad (Banerjee and Satpathy, 2025).

This study aimed to: (1) compare HRV indices across time-domain, frequency-domain, and Poincare plot measures between adults with MDD and non-depressed controls; (2) examine associations between sleep quality and HRV within each group; (3) assess whether physical activity modifies sleep–HRV relationships; and (4) explore sex-specific differences in sleep–autonomic coupling.

## Methods

### Study design and participants

This cross-sectional study was conducted at the Dr BC Roy Multi-Speciality Medical Research Centre, Indian Institute of Technology Kharagpur, West Bengal, India, from January 2024 to June 2025. Ethical approval was granted by the Institutional Ethics Committee (IIT/SRIC/DEAN/2022), and written informed consent was obtained from all participants in accordance with the Declaration of Helsinki.

Ninety-two adults aged 18–45 years were recruited using purposive sampling: 46 with DSM-5-TR–confirmed MDD and 46 non-depressed controls. Sample size was estimated *a priori* using a standard formula for comparison of two independent means, assuming a moderate effect size (d = 0.65), a significance level (α) of 0.05, and statistical power of 80%. This yielded a minimum required sample of 46 participants per group.

### Sociodemographic and clinical characteristics

Sociodemographic data including age, sex, educational status, occupation, marital status, and socioeconomic status were collected using a structured proforma. Anthropometric measurements included height, weight, and body mass index (BMI), obtained using standardised procedures. Blood pressure was recorded as the mean of two readings after a 5-minute seated rest.

### Inclusion and exclusion criteria

Inclusion criteria for the MDD group were: (1) age 18–45 years; (2) diagnosis of a current major depressive episode based on DSM-5-TR criteria confirmed through a structured clinical interview conducted by a board-certified psychiatrist; (3) a score ≥10 on the Patient Health Questionnaire-9 (PHQ-9); and (4) provision of written informed consent. The PHQ-9 ≥10 criterion was applied alongside DSM-5-TR diagnosis to ensure enrolment of participants with at least moderate current symptom severity, consistent with prior HRV research in MDD populations and to minimise heterogeneity arising from inclusion of subclinical or mild episodes.

Exclusion criteria included: current antidepressant pharmacotherapy or electroconvulsive therapy (ECT); comorbid psychotic disorders, bipolar disorder, or active substance use disorder; and known cardiovascular, metabolic, or neurological conditions that could affect autonomic function.

Absence of current or lifetime DSM-5-TR mood or anxiety disorders in the control group was confirmed using the Mini International Neuropsychiatric Interview (MINI 7.0), administered by a trained research clinician, and a PHQ-9 score <10 was required. The two groups were frequency-matched for age and sex (Grover and Laxmi, 2024).

### Assessments

Depressive symptom severity was quantified using the Patient Health Questionnaire-9 (PHQ-9) (Cronbach’s α = 0.89) (Kroenke et al., 2001). Sleep quality was assessed with the Pittsburgh Sleep Quality Index (PSQI) (Cronbach’s α = 0.83). A global PSQI score ≥ 7 was used to define poor sleep quality; while the original Buysse et al. (1989) validation proposed a threshold of > 5, a threshold of ≥ 7 has demonstrated superior discriminant validity in psychiatric populations including MDD Physical activity was evaluated using the WHO Global Physical Activity Questionnaire (GPAQ) participants were classified as inactive (<600 MET-min/week) or active (≥600 MET-min/week) per WHO guidelines. Anthropometric measures were obtained using standardised protocols; blood pressure was recorded as the mean of two readings after 5-minute seated rest (Mollayeva et al., 2016; Bull et al., 2020).

### HRV recording and analysis

Short-term HRV recordings were obtained using a PowerLab 8/35 data acquisition system (ADInstruments, Sydney, Australia). Following 5-minute supine rest, a 5-minute ECG recording was acquired using a three-lead configuration and digitised at 1 kHz. Recordings were processed using LabChart v8.1. All analyses adhered to the 1996 Task Force standards of the European Society of Cardiology (Shaffer and Ginsberg, 2017).

Time-domain measures included Standard deviation of normal-to-normal intervals (SDNN), Root mean square of successive differences (rMSSD), and Percentage of successive NN intervals differing by more than 50 ms (pNN50). Frequency-domain analysis employed Fast Fourier Transformation to yield total power, Low frequency (LF) power (0.04–0.15 Hz), and High frequency (HF) power (0.15–0.40 Hz); all frequency-domain values were natural log-transformed. Poincare plot analysis yielded standard deviations SD1, SD2, and the SD1/SD2 ratio. SD1 is mathematically equivalent to (√2/2) × rMSSD and predominantly indexes parasympathetic modulation (Bauer et al., 2017).

### Statistical analysis

Data were analysed using IBM SPSS Statistics v27. Normality was assessed by Shapiro-Wilk test. Prior to each independent-samples t-test, Levene’s test for homogeneity of variance was applied; where significant (p < 0.05), the Welch correction for unequal variances was used. Between-group differences were evaluated by independent-samples t-tests and chi-square tests. Pearson correlations quantified bivariate associations between PSQI scores and HRV parameters, stratified by group and sex. Multivariable linear regression examined independent associations between PSQI scores and each HRV parameter, adjusting for depression group, age, sex, and BMI. Prior to each regression model, the following diagnostics were performed: (1) residuals were tested for normality using the Shapiro-Wilk test; (2) multicollinearity was assessed using Variance Inflation Factors (VIF); VIF values did not exceed 2.8 across any model, indicating acceptable collinearity; (3) influential observations were identified using Cook’s distance, with no observation exceeding the threshold of 4/n. Effect modification by physical activity was assessed via stratified analysis and interaction terms. Statistical significance was defined as two-tailed α = 0.05. Given the exploratory and hypothesis-generating nature of the sex-stratified and subgroup analyses, no formal correction for multiple comparisons was applied *a priori*; however, the large number of statistical tests increases the risk of type I error, and all secondary analyses should be interpreted as hypothesis generating rather than confirmatory.

## Results

### Participant characteristics

Ninety-two participants were enrolled (MDD: n = 46; controls: n = 46). Groups were comparable for age (27.9 ± 5.6 vs 28.9 ± 6.0 years; p = 0.42) and sex distribution (56.5% male in both groups). MDD participants demonstrated significantly higher PHQ-9 scores (16.7 ± 4.2 vs 4.2 ± 2.8; p < 0.001), greater prevalence of poor sleep quality (PSQI ≥ 7: 82.6% vs 47.8%; p < 0.001), and higher rates of physical inactivity (65.2% vs 41.3%; p = 0.02). All MDD participants met DSM-5-TR diagnostic criteria and none were receiving antidepressant pharmacotherapy at enrolment. Full characteristics are in **Table 1**.

**Table 1:** Demographic and clinical characteristics.

Characteristic	Total (N = 92)	MDD (n = 46)	Control (n = 46)
Age (years), mean ± SD	28.4 ± 5.8	27.9 ± 5.6	28.9 ± 6.0
Male sex, n (%)	52 (56.5)	26 (56.5)	26 (56.5)
BMI (kg/m²), mean ± SD	23.6 ± 3.9	24.3 ± 4.2	22.9 ± 3.5
PHQ-9 score, mean ± SD	10.9 ± 6.8	16.7 ± 4.2***	4.2 ± 2.8***
Poor sleep quality (PSQI ≥ 7), n (%)	60 (65.2)	38 (82.6)***	22 (47.8)***
Global PSQI score, mean ± SD	8.62 ± 4.08	11.4 ± 3.6***	5.8 ± 3.2***
Low physical activity (<600 MET-min/week), n (%)	49 (53.3)	30 (65.2)**	19 (41.3)**
Systolic BP (mmHg), mean ± SD	115.2 ± 10.8	117.4 ± 11.3	113.0 ± 10.1
MDD episode duration (months), mean ± SD	—	8.4 ± 6.2	N/A

Data are mean ± SD or n (%). Significance markers indicate between-group comparisons: **p < 0.01; ***p < 0.001 (MDD vs Control). BMI, body mass index; BP, blood pressure; MDD, major depressive disorder; PHQ-9, Patient Health Questionnaire-9; PSQI, Pittsburgh Sleep Quality Index.

### Sleep quality components

MDD participants demonstrated significantly greater impairment across all seven PSQI components relative to controls (all p < 0.01; **Table 2**). The largest between-group differences were observed for subjective sleep quality (2.18 ± 0.72 vs 1.30 ± 0.78), sleep duration (2.04 ± 0.71 vs 1.22 ± 0.68), and daytime dysfunction (1.94 ± 0.82 vs 0.98 ± 0.81; all p < 0.001). The control group’s mean PSQI of 5.8 ± 3.2 is consistent with published normative data for young adults in South Asian university-affiliated settings and likely reflects the sleep burden of academic and professional schedules in this convenience sample; it does not indicate clinical sleep disorder.

**Table 2:** PSQI component scores by group.

PSQI component	Total (N = 92)	MDD (n = 46)	Control (n = 46)
Global PSQI score	8.62 ± 4.08	11.4 ± 3.6***	5.8 ± 3.2***
Subjective sleep quality	1.74 ± 0.84	2.18 ± 0.72***	1.30 ± 0.78***
Sleep latency	1.58 ± 0.76	1.92 ± 0.68**	1.24 ± 0.71**
Sleep duration	1.63 ± 0.79	2.04 ± 0.71***	1.22 ± 0.68***
Habitual sleep efficiency	1.42 ± 0.73	1.76 ± 0.67**	1.08 ± 0.69**
Sleep disturbances	1.65 ± 0.68	1.98 ± 0.64**	1.32 ± 0.62**
Use of sleep medication	0.74 ± 0.54	0.98 ± 0.58**	0.50 ± 0.42**
Daytime dysfunction	1.46 ± 0.92	1.94 ± 0.82***	0.98 ± 0.81***

Each component scored 0–3; global score range 0–21. Significance markers indicate between-group comparisons: **p < 0.01; ***p < 0.001 (MDD vs Control).

### HRV by depression group and sex

MDD participants exhibited significantly reduced HRV across all time-domain, frequency-domain, and Poincare plot parameters relative to controls (**Table 3**). In the time domain, SDNN was 32.4% lower in MDD (39.2 ± 14.8 vs 58.0 ± 18.6 ms; p < 0.001), rMSSD was 40.7% lower (27.4 ± 12.6 vs 46.2 ± 15.4 ms; p < 0.001), and pNN50 similarly attenuated (7.8 ± 6.4 vs 17.0 ± 10.2%; p < 0.001). Frequency-domain analysis revealed reduced total power, LF power, and HF power, with an elevated LF/HF ratio (3.42 ± 1.28 vs 2.26 ± 0.86; p < 0.01) consistent with sympathetic predominance.

**Table 3:** HRV measures by group and sex.

HRV parameter	Total (N = 92)	MDD (n = 46)	Control (n = 46)
Average HR (bpm)	76.4 ± 11.8	80.2 ± 12.4**	72.6 ± 10.2**
Time domain			
SDNN (ms)	48.6 ± 18.4	39.2 ± 14.8***	58.0 ± 18.6***
rMSSD (ms)	36.8 ± 16.2	27.4 ± 12.6***	46.2 ± 15.4***
pNN50 (%)	12.4 ± 9.6	7.8 ± 6.4***	17.0 ± 10.2***
Poincare plot			
SD1 (ms)	26.0 ± 11.6	19.4 ± 8.8***	32.6 ± 11.2***
SD2 (ms)	64.8 ± 22.4	52.2 ± 18.6***	77.4 ± 21.8***
SD1/SD2 ratio	0.40 ± 0.09	0.37 ± 0.08*	0.42 ± 0.09*
Frequency domain			
Total power (ln ms²)	7.62 ± 0.82	7.18 ± 0.76***	8.06 ± 0.72***
LF power (ln ms²)	6.48 ± 0.74	6.06 ± 0.70***	6.90 ± 0.66***
HF power (ln ms²)	5.96 ± 0.92	5.36 ± 0.88***	6.56 ± 0.80***
LF/HF ratio	2.84 ± 1.12	3.42 ± 1.28**	2.26 ± 0.86**
Males (n = 52)			
SDNN (ms)	52.4 ± 19.2	42.6 ± 15.4**	62.2 ± 18.8**
SD1 (ms)	28.6 ± 12.2	21.8 ± 9.2**	35.4 ± 11.6**
SD2 (ms)	70.2 ± 24.6	56.8 ± 20.4**	83.6 ± 22.2**
Females (n = 40)			
SDNN (ms)	43.8 ± 16.4	34.2 ± 12.6**	53.4 ± 16.2**
SD1 (ms)	22.8 ± 10.4	16.2 ± 7.8**	29.4 ± 10.0**
SD2 (ms)	57.6 ± 18.8	45.8 ± 15.6**	69.4 ± 18.4**

Significance markers indicate between-group comparisons: *p < 0.05; **p < 0.01; ***p < 0.001 (MDD vs Control). HF, high frequency; LF, low frequency; pNN50, proportion of NN intervals > 50 ms; rMSSD, root mean square of successive differences; SD1/SD2, Poincare plot indices; SDNN, standard deviation of NN intervals.

Poincare plot analysis further differentiated the groups. SD1 was 40.5% lower in MDD (19.4 ± 8.8 vs 32.6 ± 11.2 ms; p < 0.001) and SD2 was 32.5% lower (52.2 ± 18.6 vs 77.4 ± 21.8 ms; p < 0.001). The SD1/SD2 ratio was reduced in MDD (0.37 ± 0.08 vs 0.42 ± 0.09; p < 0.05). Sex-stratified analyses indicated that between-group HRV differences were more pronounced in females than in males across all parameters.

### Associations between sleep quality and HRV

Across the total sample, higher PSQI scores were inversely correlated with all HRV parameters, with the strongest associations for SD1 (r = −0.322; p < 0.01), rMSSD (r = −0.318; p < 0.01), and HF power (r = −0.312; p < 0.01; **Table 4**). Group-stratified analyses revealed substantially stronger correlations in MDD participants: HF power (r = −0.524), SD1 (r = −0.528), SD2 (r = −0.504), SDNN (r = −0.476; all p < 0.01). In non-depressed controls, correlations were weak. Sex-stratified analyses identified females with MDD as the highest-risk subgroup (SD1: r = −0.692; HF power: r = −0.684; both p < 0.01).

**Table 4:** Pearson correlations between PSQI global scores and HRV parameters.

Subgroup	n	HF (ln)	LF (ln)	SDNN	rMSSD	SD1	SD2
Total	92	−0.312**	−0.274**	−0.286**	−0.318**	−0.322**	−0.294**
Males	52	−0.274**	−0.248*	−0.252*	−0.278**	−0.286**	−0.260*
Females	40	−0.428**	−0.386**	−0.374**	−0.418**	−0.432**	−0.402**
MDD group	46	−0.524**	−0.498**	−0.476**	−0.512**	−0.528**	−0.504**
Control	46	−0.186*	−0.168	−0.174	−0.192*	−0.196*	−0.178
MDD males	26	−0.384**	−0.348*	−0.338*	−0.368**	−0.376**	−0.352*
MDD females	20	−0.684**	−0.626**	−0.598**	−0.648**	−0.692**	−0.662**
Control males	26	−0.162	−0.148	−0.156	−0.174	−0.178	−0.158
Control females	20	−0.248*	−0.224	−0.218	−0.238*	−0.244*	−0.228

*p < 0.05; **p < 0.01. HF, high frequency; LF, low frequency; rMSSD, root mean square of successive differences; SD1/SD2, Poincare plot indices; SDNN, standard deviation of NN intervals.

### Regression analysis

Multiple linear regression models (**Table 5**) demonstrated that both depression group and PSQI scores were independently associated with reduced HRV after adjusting for age, sex, and BMI (all models p < 0.01; adjusted R² = 0.148–0.204). MDD group membership independently predicted lower HF power (β = −0.748), rMSSD (β = −12.84 ms), SD1 (β = −8.94 ms), SD2 (β = −18.62 ms), and SDNN (β = −16.48 ms). Each one-point PSQI increment was independently associated with decrements in all HRV parameters. Female sex was consistently associated with lower HRV (all p < 0.05).

**Table 5:** Multivariable regression predicting HRV from depression group and sleep quality, adjusted for age, sex, and BMI.

HRV parameter	Predictor	β (SE)	95% CI	p-value	Adj. R²
HF power (ln ms²)	MDD vs Control	−0.748 (0.214)	[−1.170, −0.326]	0.001	0.186
	Female sex	−0.362 (0.158)	[−0.672, −0.052]	0.024	
	PSQI score	−0.224 (0.076)	[−0.373, −0.075]	0.004	
rMSSD (ms)	MDD vs Control	−12.84 (4.26)	[−21.21, −4.47]	0.003	0.162
	Female sex	−8.62 (3.12)	[−14.74, −2.50]	0.007	
	PSQI score	−4.86 (1.48)	[−7.77, −1.95]	0.001	
SD1 (ms)	MDD vs Control	−8.94 (2.86)	[−14.55, −3.33]	0.002	0.178
	Female sex	−5.74 (2.08)	[−9.83, −1.65]	0.007	
	PSQI score	−1.62 (0.52)	[−2.64, −0.60]	0.002	
SD2 (ms)	MDD vs Control	−18.62 (6.48)	[−31.33, −5.91]	0.005	0.148
	Female sex	−10.84 (4.72)	[−20.10, −1.58]	0.023	
	PSQI score	−3.28 (1.12)	[−5.48, −1.08]	0.004	
SDNN (ms)	MDD vs Control	−16.48 (5.62)	[−27.51, −5.45]	0.004	0.204
	Female sex	−18.26 (4.08)	[−26.27, −10.25]	<0.001	
	PSQI score	−3.84 (1.86)	[−7.49, −0.19]	0.042	

β, unstandardised coefficient; Adj. R², adjusted R-squared; SE, standard error; CI, confidence interval.

### Physical activity as a modifier

Significant interaction effects between physical activity level and sleep quality were observed for HF power (p < 0.001), SD1 (p = 0.002), and SDNN (p = 0.006; **Table 6**). Among physically inactive participants, poor sleep was associated with substantially greater HRV reductions than among active participants, demonstrating a partial protective association of regular physical activity against sleep-related autonomic deterioration.

**Table 6:** HRV stratified by physical activity and sleep quality.

Physical activity	Sleep quality	n	HF power (ln ms²)	SD1 (ms)	SDNN (ms)
Low (<600 MET-min/wk)	Good (PSQI < 7)	16	5.72 ± 0.82	20.8 ± 8.4	50.2 ± 16.8
	Poor (PSQI ≥ 7)	30	4.98 ± 0.88**	15.6 ± 7.2**	38.4 ± 14.6**
Moderate/vigorous (≥600)	Good (PSQI < 7)	27	6.88 ± 0.78	33.6 ± 10.8	62.4 ± 18.2
	Poor (PSQI ≥ 7)	19	6.32 ± 0.86*	28.4 ± 9.6*	52.8 ± 16.4*
Interaction			p < 0.001	p = 0.002	p = 0.006

*p < 0.05; **p < 0.01 vs good sleep within same activity stratum. HF, high frequency power; PSQI, Pittsburgh Sleep Quality Index; SD1, short-axis Poincare standard deviation; SDNN, standard deviation of NN intervals.

## Discussion

This cross-sectional study provides multidimensional evidence that MDD is associated with markedly reduced cardiac autonomic modulation in young adults, assessed through time-domain, frequency-domain, and Poincare plot HRV measures. MDD participants exhibited significantly reduced HRV across all domains; poor sleep quality was highly prevalent in MDD and was associated with further HRV reductions; Poincare plot analysis revealed acute parasympathetic suppression (reduced SD1) and impaired long-term autonomic regulation (reduced SD2); sleep–HRV correlations were substantially stronger in MDD, and most pronounced in females with MDD; and physical activity was associated with modification of sleep–HRV relationships.

In the present study, MDD participants demonstrated a 40.5% reduction in SD1 relative to controls. The observed SDNN of 39.2 ms in the MDD group falls within ranges associated with compromised cardiac health and elevated cardiovascular risk. The Poincare plot data add clinically important resolution: the substantially reduced SD1 (19.4 vs 32.6 ms) is consistent with acute parasympathetic withdrawal, while reduced SD2 (52.2 vs 77.4 ms) reflects impaired long-term autonomic regulation. The reduced SD1/SD2 ratio (0.37 vs 0.42) is consistent with sympathetic predominance in MDD, a pattern associated with chronic HPA axis activation and attenuated vagal tone characteristic of the disorder. These findings align closely with the largest available meta-analytic benchmarks: Koch et al., pooling 21 unmedicated MDD cohorts (N = 4,235), reported Hedges’ g = −0.46 for RMSSD, −0.27 for SDNN, −0.32 for HF power, and +0.20 for LF/HF ratio, effect sizes consistent with the present between-group differences; the umbrella review by Kreppke et al., spanning 90 meta-analyses, further confirmed that nonlinear HRV abnormalities in MDD carry even larger effect sizes (g ≥1.03), underscoring that the Poincare plot indices reported here may capture autonomic dysregulation that conventional time- and frequency-domain parameters alone underestimate (Thayer et al., 2010; Chen et al., 2017; Koch et al., 2019; Kreppke et al., 2026).

The following mechanistic pathway is proposed on the basis of convergent evidence from prior experimental and longitudinal research and should be understood as interpretive in the context of the present cross-sectional design, as cortisol levels, inflammatory markers, and direct sympathetic activity were not measured in this study. The high prevalence of poor sleep in MDD (82.6% vs 47.8%) is consistent with established epidemiological data. The independent association of PSQI scores with reduced HRV, retained after controlling for depression group, age, sex, and BMI, is consistent with a direct mechanistic pathway through which sleep quality may influence autonomic regulation. Sleep deprivation has been associated with HPA axis activation, elevated sympathetic tone, and increased circulating inflammatory cytokines, each of which may contribute to reduced vagal activity (Trinder et al., 2001; Sforza et al., 2007; Chien et al., 2015a). Convergent evidence from neurobiological reviews suggests that sleep disturbance in depression involves dysfunction in prefrontal-limbic autonomic circuits that may directly impair cardiac vagal modulation (Guerrera et al., 2024).

The markedly stronger sleep–HRV correlations in MDD, particularly for SD1 (r = −0.528 vs −0.196 in controls), suggest heightened autonomic sensitivity to sleep quality fluctuations in depressed individuals, likely reflecting reduced autonomic resilience and narrowed regulatory range. This is consistent with evidence that sleep-related psychopathology in mood disorders operates through overlapping neurobiological pathways involving the reticular activating system and hypothalamic sleep–wake centres. This positions sleep as a high-leverage intervention target in MDD (Lin et al., 2019).

Sex differences in sleep–autonomic coupling warrant specific attention. Females demonstrated lower absolute HRV values than males across both groups, and the sleep–HRV associations were substantially stronger among females with MDD (SD1 r = −0.692). These patterns identify females with MDD as a high-risk subgroup requiring targeted cardiovascular autonomic surveillance. The buffering effect of physical activity on sleep–HRV associations is clinically actionable. Among physically active participants, HRV decrements associated with poor sleep were substantially attenuated. Exercise has been associated with improved parasympathetic tone through baroreflex sensitisation, reduced neuroinflammation, and stabilisation of circadian rhythms mechanisms that may partially explain the observed attenuation of sleep-related HRV decrements in active participants. Given that 65.2% of MDD participants were physically inactive, physical activity promotion should be regarded as integral rather than adjunctive in MDD management (Chien et al., 2015b; Chu et al., 2017; Pearce et al., 2022; Singh et al., 2023).

### Strengths

The study employed a comprehensive multidomain HRV assessment incorporating time-domain, frequency-domain, and nonlinear Poincare plot indices, thereby capturing autonomic dysregulation at a level of detail rarely achieved in previous studies. A major strength is the inclusion of a medication-naïve MDD cohort, eliminating antidepressant-related pharmacological confounding of HRV, a limitation highlighted in prior reviews. Another notable strength is the simultaneous evaluation of sleep quality, physical activity, and HRV within the same cohort, addressing an important gap identified in umbrella reviews and further strengthened by the analysis demonstrating physical activity as a significant modifier of sleep-related autonomic dysfunction.

### Limitations

The cross-sectional design precludes causal inference. The sample size of 46 per group limits statistical power for subgroup analyses. The multiple statistical comparisons conducted across subgroups and HRV parameters increase the risk of type I error; future confirmatory studies should pre-register primary and secondary outcomes and apply appropriate corrections (e.g., Bonferroni or false discovery rate). Several variables known to influence HRV were not systematically assessed or controlled, including smoking status, caffeine and alcohol intake, occupational stress levels, menstrual cycle phase and hormonal contraceptive use in female participants, and antihypertensive or other non-antidepressant medications. Hormonal status is particularly relevant given the sex-specific findings, as menstrual cycle phase is known to modulate cardiac vagal tone. The observed between-group and sex-specific HRV differences may therefore partly reflect unmeasured confounding and should be interpreted with appropriate caution. Reliance on the PSQI as a self-report measure represents a notable limitation. Although the PSQI is extensively validated and widely used in both research and clinical settings, it is susceptible to recall bias and cannot capture objective sleep architecture, sleep staging, or night-to-night variability. Future studies should incorporate objective sleep assessment using validated wrist actigraphy or polysomnography, which would allow examination of specific sleep architecture parameters particularly slow-wave sleep and REM sleep, both implicated in cardiac autonomic regulation in relation to HRV. The exclusion of participants receiving antidepressant pharmacotherapy, while necessary to minimise HRV confounding, limits generalisability to the broader treated MDD population.

## Conclusion

Major depressive disorder is independently and markedly associated with reduced cardiac autonomic modulation across time-domain, frequency-domain, and Poincare plot HRV indices. Poor sleep quality, highly prevalent in MDD, is independently associated with additional HRV attenuation, with particularly strong associations in females with MDD. Adequate physical activity partially buffers sleep-related autonomic deterioration. These cross-sectional findings are consistent with the hypothesis that comprehensive HRV assessment, systematic sleep quality screening, and structured physical activity promotion may be beneficial components of MDD management. Longitudinal interventional trials are warranted to evaluate whether sleep and exercise interventions can improve autonomic function and reduce cardiovascular mortality risk in this vulnerable population.

## Data Availability

The original contributions presented in the study are included in the article/supplementary material, further inquiries can be directed to the corresponding author.
